# Pesticide safety training to promote sustainable practices among French tree fruit and fresh vegetable farmers: A pre-post intervention survey

**DOI:** 10.1371/journal.pone.0328161

**Published:** 2025-07-18

**Authors:** Sonia Grimbuhler, Jean-François Viel

**Affiliations:** 1 INRAE, National Research Institute for Agriculture, Food and Environment, ITAP Research Team “Technologies and Methods for the Agriculture of Tomorrow”, Institut Agro, Univ Montpellier, Montpellier, France; 2 Univ Rennes, CHU Rennes, Inserm, EHESP, Irset (Institut de Recherche en Santé, Environnement et Travail) - UMR_S 1085, Rennes, France; Lusofona University of Humanities and Technologies: Universidade Lusofona de Humanidades e Tecnologias, PORTUGAL

## Abstract

**Objective:**

Reducing pesticide exposure in agricultural activities remains a major challenge, particularly for crops that rely heavily on pesticides, such as fruits and vegetables. Integrated pest management technology requires a strong safety culture to effectively reduce pesticide use. The aim of this study is to assess the impact of mandatory safety training on the knowledge and perceptions of pesticide use among French fruit and vegetable farmers and farm workers.

**Methods:**

Farmers throughout France were approached during mandatory training for pesticide-related activities. Trainees were asked to complete a self-administered questionnaire that included demographic and occupational data as well as a safety climate scale specifically designed for the agricultural sector. A total of 182 farm managers or workers involved in fruit cultivation and 164 involved in vegetable cultivation completed the questionnaire at the beginning and end of the session, enabling us to compare pre- and post-training scores using a multiple measures design. Statistical analyses were conducted using paired *t*-tests and mixed models for repeated measures.

**Results:**

We noted increases in global safety climate scores (8.2% and 8.6% among fruit and vegetable producers, respectively; p < 0.001) and each of their seven dimension scores after trainees completed the course. The largest increases among fruit producers were observed in the communication and feedback, and the knowledge dimensions (16.6% and 8.6%, respectively; p < 0.001). Among vegetable producers the greatest improvements were found in the communication and feedback, and the teamwork climate dimensions (14.9% and 9.4%, respectively; p < 0.001). Score improvements remained highly significant in multivariate analyses, as few covariate-by-time interactions were found.

**Conclusions:**

This study demonstrates that pesticide training significantly enhances the safety climate perception among French fruit and vegetable producers. The long-term effects of this intervention should be evaluated, with the ultimate goal of reducing risks to human health and the environment.

## Introduction

Reducing pesticide exposure in agricultural activities remains a major challenge, particularly for crops that rely heavily on pesticides. A critical concern involves the protection of rural residents who may be subjected to repeated, long-term exposure to pesticides due to spray drift from surrounding crop fields [1]. Additionally, pesticide applications during fruit and vegetable development may result in residues that are traceable after harvest, which are among European Union (EU) citizens’ predominant food-related concerns, as diet is humans’ main source of exposure to pesticides [2]. Compared to other crops, fruits and vegetables receive a considerably higher amount of pesticides due to the significant presence of pests, diseases, and weeds, which can lead to substantial yield losses if left untreated [3]. Moreover, strict rules dictate the acceptable amount of damage on fruits and vegetables to meet consumer demand for blemish-free products, which maintains the incentive for intense pesticide use.

At the EU level, France ranks fifth in fruit production and third in vegetable production [4]. The corresponding specialized farms cover 129,000 and 53,000 hectares, and engage 33,230 and 53,060 full-time equivalents (FTEs), respectively [5]. Although a wide variety of fruits are cultivated, apples and peaches are by far the most pesticide-intensive crops, with 35.9 and 21.2 mean applications per year, respectively [6]. Vegetable crops receive a lower but still high quantity of pesticides, with mean applications per year of 13.6 for carrots and 13.0 for tomatoes [7]. This vulnerability to a wide variety of pests and diseases is one of the primary reasons for the labor-intensive nature of fruit and vegetable farming compared to other agricultural sectors.

To reduce the adverse effects of chemical pesticides on human health and the environment, the EU aims to decrease reliance on pesticides in agriculture by implementing more integrated and sustainable approaches while ensuring stable agricultural production. These principles were incorporated into EU policy through Directive 2009/128/EC on the sustainable use of pesticides (Article 14) [8]. Integrated pest management technology (substituting synthetic chemicals with alternative practices, enhancing plant protection by using decision-support tools and modern precision agriculture systems, redesigning production systems to focus on biodiversity, and promoting plant defenses through genetics or cultural practices) [9], requires a strong safety culture to effectively reduce pesticide use. One key element of the general safety culture, which embodies the value placed on safety and the extent to which people assume personal responsibility for organizational safety, is training and qualification to improve farmers’ skills [10]. Continuous training empowers farmers with the knowledge, skills, and resources needed to strengthen their belief in pesticide hazard control and adopt better safety behaviors and more sustainable pest management practices [11–13]. These new behaviors and practices can lead to a reduction in pesticide use in several ways. Improved knowledge and awareness can help farmers identify pests accurately, understand their life cycles, and apply more effective, targeted control methods. Adoption of sustainable practices, such as crop rotation, biological controls, and the use of resistant crop varieties, can reduce pest populations naturally. Continuous education equips farmers with the skills to make better decisions and apply pesticides only when necessary and in appropriate quantities, reducing overuse. Finally, training sessions provide opportunities for farmers to share experiences and solutions with peers. This community support can reinforce positive changes in pesticide use and encourage the adoption of best practices.

Training evaluations can provide valuable insights into its effects, thereby enabling agricultural authorities and instructors to design more effective training components. However, there is surprisingly limited evidence to support the efficacy of training designed to improve knowledge, beliefs, practices, behaviors, and perceptions related to reducing pesticide exposure among farmers and farmworkers. Afshari et al. selected and reviewed 31 studies, all conducted outside of Europe [14]. The majority of these studies (21 out of 31) focused on educational and behavioral interventions, while the remaining interventions included incentives, engineering and technology, legislation and enforcement, or multifaceted programs. The outcomes were primarily assessed through self-reported data, but eight articles used biological measures to evaluate the effects of interventions. The authors found that educational interventions effectively improved the knowledge and attitudes of participants, but were less successful in changing behavior and, consequently, in reducing exposure to toxic pesticides.

Ayaz et al. subsequently performed a meta-analysis of 38 studies focusing on educational interventions, also all conducted outside of Europe [15]. While structured or semi-structured measurement tools were generally used, in seven studies, the Knowledge, Attitudes, and Practices (KAP) score was calculated, in one study, the Pesticide Knowledge Test was applied, and in one study, the Risk Behavior Diagnosis Scale was implemented. The authors demonstrated that educational interventions had a significant impact on the knowledge of agricultural workers, a moderate effect on their behavior, and a minor effect on risk perception. It was found that studies grounded in theory and organized with repeated sessions had the greatest impact on both knowledge and behavior levels. Similarly, studies utilizing multicomponent educational methods showed a significant effect on behavior levels.

In addition to the studies included in these review papers, we identified four more studies that were of interest. In Northern Greece, in areas where mainly cotton is cultivated, most trained farmers have a higher level of knowledge regarding pesticide use, stronger beliefs in pesticide hazard control, and better safety behavior compared to non-trained farmers [13]. Cevik et al. observed that training Turkish farmers in the safe use of pesticides encouraged them to develop positive behavioral changes [16]. Rattanawitoon et al. found a significant improvement in knowledge, attitudes, practices, and beliefs about chemical pesticide exposure among female farmworkers in Thailand after participating in a pesticide training program [17]. Grimbuhler et al. showed that compulsory training in pesticide safety is highly effective in improving safety climate perception among French cereal farmers [18].

Pesticide use varies across regions due to differences in climate, pests, crops, farming systems, technologies, regulations, economic realities, culture, education, and public pressure. These factors influence the reasons, methods, and types of pesticides that are used. Therefore, the transferability of training effectiveness from one region to another cannot be assumed. Existing evidence on the effectiveness of interventions to promote pesticide safety reveals research gaps particularly in Europe and on the most pesticide-intensive crops. Therefore, the aim of this study is to assess the impact of mandatory safety training on the perceptions of French fruit and vegetable farmers regarding pesticide safety procedures and practices. It is assumed that safety climate scores will increase from the start to the end of the training course as the result of the training content and group dynamics.

## Population and methods

### Pesticide safety training

To comply with European legislation, France has developed a national action plan to implement the guidelines outlined in Directive 2009/128/EC and its Annex 1 [8]. One of the measures included is the requirement for farmers to possess a certificate, known as *Certiphyto*, to use and purchase phytosanitary products. This credential is earned through mandatory training as part of the authorization process, which outlines the criteria for awarding, renewing, and revoking certificates. Certificates are initially awarded for a period of five years after completing a two-day training program. The *Certiphyto* training program has been consistently improved with the knowledge acquired from recent research, technological advancements, and new regulations in place. It covers various aspects such as pesticide use legislation, hazards and risks associated with pesticides, measures to minimize risks to humans, non-target organisms and the environment [19]. It also includes integrated pest management strategies and techniques, comparative assessments at the user level to assist professional users in making the most appropriate choices on pesticides, farm-level occupational and environmental risk assessments, and equipment as well as working practices management to reduce exposure to pesticides. After a five-year period, farmers must attend a one-day refresher course to update their knowledge. Furthermore, *Certiphyto* training is specifically designed for two sets of participants — decision-makers and operators — to ensure appropriate pesticide use.

### Study population

Farmers throughout France were approached during their mandatory *Certiphyto* training to request their participation in this study. The study population considered here comprised farm managers and workers attending either the initial *Certiphyto* training course or a refresher course. The trainers from 125 *Certiphyto* sessions (organized between March 2021 and April 2023 throughout France) agreed to allocate some of the training time to allow participants to complete the survey. In total, 1,411 trainees participated in this study and completed a standardized self-administered questionnaire twice: at the start (D0) and end (D1 for one-day refresher courses or D2 for two-day initial courses, without access to their D0 responses) of the training session.

However, *Certiphyto* training courses are organized for all farmers who come into contact with pesticides regardless of their agricultural activities. For this study, we focused on participants who are active in fruit or vegetable farming. In total, 217 farm managers or workers involved in fruit cultivation completed the safety climate questionnaire on D0, with 35 of them declining to complete the questionnaire again on D1 or D2. For vegetable cultivation, 30 out of the 194 participants who completed the safety climate questionnaire on D0 chose not to complete it at the end of the course. This resulted in a total of 182 farm managers or workers involved in fruit cultivation and 164 involved in vegetable cultivation with paired and complete responses for pre-training and post-training comparisons.

### Safety climate scale

The safety climate is a measurable proxy for the prevailing safety culture [20]. Many authors have defined a safety climate based on the shared perception of security concerns (refer to the review by Luo [21]); accordingly, we also view safety climate as the combination of shared perceptions among workers regarding procedures, practices, attitudes, and behaviors related to occupational safety [10]. We used a self-administered safety climate questionnaire that was specifically designed and validated for the agricultural sector [22,23]. This scale focuses on individuals’ perceptions regarding pesticide use, safety, and regulations. The reliability (Cronbach’s alpha of 0.81) and construct validity of this scale have been shown to be good. The psychometric model consists of seven dimensions as determined through confirmatory analysis using structural equation modeling. These dimensions (management commitment, communication and feedback, rules and best practices, knowledge, safety compliance, safety participation, and teamwork climate) are evaluated through 20 items. Each of the 20 items is rated on a 5-point Likert scale. These scores are then summed to obtain a global score ranging from 20 to 100, with higher scores indicating a better safety climate.

### Data collection

Given the limited research on safety climate in agriculture, and our focus on pesticide-related safety practices, this study relied on a conceptual model that incorporates demographic and farm characteristics known to be associated with occupational injuries and unsafe conditions [24], as well as pesticide use. The first section of the self-administered questionnaire covers individual and occupational data meeting these conditions: sex, age, experience in pesticide use, position within the farm, type of certification, pesticide-related activities, type of agricultural system, and agricultural cooperative membership. The second section includes the safety climate scale itself. The data was collected either on paper forms or using an online survey tool, depending on the availability of computer workstations at the training site. Participants provided verbal informed consent, which was witnessed by the session trainer, who was not part of the study team. As the questionnaire for this perception survey did not contain any health-related data and was completed anonymously during a mandatory certification procedure for training content assessment, no ethics committee approval was required according to the European Union regulations in force [25].

### Statistical analyses

We considered the *Certiphyto* training course to be an intervention that would likely lead to a change in scores, as the safety climate is known to vary over time and in different circumstances [21]. Therefore, this study was based on a repeated measures design in which the subjects acted as their own controls. In statistical terms, the error term contains only the variability within subjects and not the variability between subjects, making this design well-suited for assessing effects over time (such as post-intervention) with increased statistical power.

To compare the mean global scores obtained before and after training (for the 182 and 164 participants with paired scores), we initially used a univariate approach based on paired *t*-tests. Cohen’s d for paired samples was used to describe the standardized mean difference and assess the magnitude of the intervention [26]. We then tested the differences in safety climate global scores with a mixed model for repeated measures (MMRM) [27], which included terms for risk factors, time (D0 and D1 or D2), and risk factor-by-time interactions. Covariate-by-time interactions (p < 0.20) that were identified in univariate analyses were included in multivariate MMRMs [28]. We then performed analyses for each sub-score to guide the further development and implementation of more effective interventions. We considered p-values < 0.05 to be statistically significant, and all tests were two-tailed. Statistical analyses were performed with the base and mmrm packages of R software [29,30].

## Results

### Survey participants

Demographic and occupational data for the farm managers and workers who participated in the safety climate survey are presented in Table 1. The distribution of demographic and occupational data is quite similar between participants for the two types of cultivation. The majority of respondents were male, under 50 years of age, had less than 10 years of experience in pesticide use, were engaged in all pesticide-related activities, practiced integrated or organic farming, and were not affiliated with an agricultural cooperative.

**Table 1:** Demographic characteristics of farmers and farm workers, and occupational factors.

	Fruit farmers (n = 182)		Vegetable farmers (n = 164)	
	Number	Percentage	Number	Percentage
**Sex**				
Female	39	21.4%	57	34.8%
Male	143	78.6%	107	65.2%
**Age (years)**				
< 40	83	45.6%	84	51.2%
40-49	46	25.3%	35	21.3%
50-59	34	18.7%	29	17.7%
≥ 60	19	10.4%	16	9.8%
**Experience in pesticide use (years)**				
< 5	84	46.2%	87	53.0%
5-9	15	8.2%	9	5.5%
10-14	20	11.0%	16	9.8%
≥ 15	63	34.6%	52	31.7%
**Position within the farm**				
Apprentice/student	32	17.6%	32	19.5%
Farm worker	55	30.2%	42	25.6%
Farm manager	95	52.2%	90	54.9%
**Type of certification**				
Initial	100	54.9%	99	60.4%
Renewal	82	45.1%	65	39.6%
**Pesticide-related activities**				
Decision-making				
No	47	25.8%	36	22.0%
Yes	135	74.2%	128	78.0%
Preparation				
No	51	28.0%	49	29.9%
Yes	131	72.0%	115	70.1%
Application				
No	54	29.7%	50	30.5%
Yes	128	70.3%	114	69.5%
Equipment cleaning				
No	47	25.8%	48	29.3%
Yes	135	74.2%	116	70.7%
**Type of agricultural system**				
Conventional	54	29.7%	46	28.1%
Integrated	65	35.7%	65	39.6%
Organic	63	34.6%	53	32.3%
**Agricultural cooperative membership**				
No	138	75.8%	134	81.7%
Yes	44	24.2%	30	18.3%

### Safety climate scores (univariate analyses)

The average safety climate baseline score for fruit producers was 83.41 (standard deviation [SD] = 10.73), which increased to 90.24 (SD = 9.05) after completing the *Certiphyto* course (8.2% increase; p < 0.001) (Table 2). A similar trend was seen for each of the seven dimensions (all p-values < 0.001), with improvements ranging from 3.4% in rules and best practices, and 6.7% in management commitment to 8.6% in knowledge, and 16.6% in communication and feedback. The Cohen’s d value was 0.78 for the global score indicating a rather large effect size, while the values for the subscores ranged from 0.31 to 0.83.

**Table 2:** Change in safety climate scores in response to *Certiphyto* training (univariate analysis).

	Pre-training score	Post-training score	Absolute difference	Relative difference	Cohen’s d	p-value
**Fruit tree cultivation (n = 182)**						
Global score	83.41	90.24	6.83	8.2%	0.78	< 0.001
*Management commitment*	25.23	26.91	1.68	6.7%	0.60	< 0.001
*Communication and feedback*	11.75	13.70	1.95	16.6%	0.83	< 0.001
*Rules and best practices*	9.01	9.32	0.31	3.4%	0.37	< 0.001
*Knowledge*	12.66	13.75	1.09	8.6%	0.48	< 0.001
*Safety compliance*	12.50	13.54	1.04	8.3%	0.50	< 0.001
*Safety participation*	4.31	4.55	0.24	5.6%	0.31	< 0.001
*Teamwork climate*	7.95	8.45	0.50	6.3%	0.32	< 0.001
**Fresh vegetable cultivation (n = 164)**						
Global score	84.37	91.14	6.77	8.0%	0.81	< 0.001
*Management commitment*	25.53	27.08	1.55	6.1%	0.55	< 0.001
*Communication and feedback*	11.94	13.72	1.78	14.9%	0.76	< 0.001
*Rules and best practices*	9.21	9.45	0.24	2.6%	0.26	0.001
*Knowledge*	12.74	13.92	1.18	9.3%	0.73	< 0.001
*Safety compliance*	12.81	13.74	0.93	7.3%	0.48	< 0.001
*Safety participation*	4.26	4.61	0.35	8.2%	0.49	< 0.001
*Teamwork climate*	7.88	8.62	0.74	9.4%	0.42	< 0.001

A similar pattern was observed among vegetable producers, whose mean safety climate score improved from 84.37 (SD = 9.85) at baseline to 91.14 (SD = 8.90) at the end of the *Certiphyto* course, indicating an 8.0% increase (p < 0.001) (Table 2). This trend was consistent across all seven dimensions (all p-values < 0.01), with increases ranging from 2.6% in rules and best practices, and 6.1% in management commitment to 9.4% in team climate, and 14.9% in communication and feedback. A Cohen’s d value of 0.81 was found for the global score reflecting a large effect size, while the values for the subscores ranged from 0.26 to 0.76.

### Safety climate scores (multivariate analyses)

All score changes remained statistically significant, and few covariate-by-time interactions appeared significant in multivariate analyses. Regarding fruit tree cultivation, the following risk factor-by-time interactions were identified (Table 3): age in the management and commitment dimension (40−49 years: β = 1.17, p = 0.024; 50−59 years: β = 1.53, p = 0.012; ≥ 60 years: β = 2.00, p = 0.007); type of certification in the global score (β = −5.44, p = 0.007), the management and commitment dimension (β = −1.76, p = 0.006), and the communication and feedback dimension (β = −1.58, p = 0.004); farm worker position in the knowledge dimension (β = −1.07, p = 0.046) as well as the safety participation dimension (β = −0.42, p = 0.021); and decision-making in the teamwork climate dimension (β = 0.61, p = 0.020).

**Table 3:** Results of a mixed model for repeated measures comparing pre- and post-training safety climate scores among fruit producers (multivariate analysis, n = 182).

	p*-*value^a^	Risk factor-by-time interactions (identified in univariate analyses with p < 0.20)
Global score	< 0.001	Sex, experience in pesticide use, position, type of certification**, decision making, preparation, agricultural cooperative membership
Dimensions		
*Management commitment*	< 0.001	Sex, age*, experience in pesticide use, type of certification**, preparation, agricultural cooperative membership
*Communication and feedback*	< 0.001	Experience in pesticide use, position*, type of certification**, decision making, preparation, type of agricultural system, agricultural cooperative membership
*Rules and best practices*	< 0.001	Sex, preparation
*Knowledge*	< 0.001	Experience in pesticide use, position*, type of certification, decision-making
*Safety compliance*	< 0.001	Experience in pesticide use, position, type of certification, preparation, application, equipment cleaning, agricultural cooperative membership
*Safety participation*	< 0.001	Position*, type of certification, decision making
*Teamwork climate*	< 0.001	Decision making*, application

Note. ^a^ The p-value refers to the difference between the mean scores before and after the intervention

* p* *< 0.05, ** p* *< 0.01

For fresh vegetable cultivation, the significant covariate-by-time interactions were slightly different (Table 4): type of certification in the global score (β = −3.63, p = 0.012), the communication and feedback dimension (β = −1.28, p = 0.030), and the knowledge dimension (β = −1.13, p = 0.006); farm worker position in the safety participation dimension (β = −0.36, p = 0.032); decision-making in the safety participation dimension (β = −0.37, p = 0.010); and organic agricultural system in the communication and feedback dimension (β = 1.49, p = 0.003) as well as the knowledge dimension (β = 0.32, p = 0.025).

**Table 4:** Results of a mixed model for repeated measures comparing pre- and post-training safety climate scores among vegetable producers (multivariate analysis, n = 164).

	p*-*value^a^	Risk factor-by-time interactions (identified in univariate analyses with p < 0.20)
Global score	< 0.001	Sex, position, type of certification*, decision making, preparation, application, equipment cleaning, type of agricultural system
Dimensions		
*Management commitment*	< 0.001	Type of certification
*Communication and feedback*	< 0.001	Sex, experience in pesticide use, position, type of certification*, preparation, application, equipment cleaning, type of agricultural system**
*Rules and best practices*	0.001	Agricultural cooperative membership
*Knowledge*	< 0.001	Experience in pesticide use, position, type of certification**, equipment cleaning, type of agricultural system*
*Safety compliance*	< 0.001	Sex, position, type of certification, decision making, preparation, application, equipment cleaning
*Safety participation*	< 0.001	Position*, decision making*, preparation, application, equipment cleaning
*Teamwork climate*	< 0.001	Preparation, equipment cleaning

Note. ^a^ The p-value refers to the difference between the mean scores before and after the intervention.

* p* *< 0.05, ** p* *< 0.01

## Discussion

This study demonstrates that pesticide training significantly enhances the safety climate among farmers and farm workers who use pesticides extensively, which provides further support for the effectiveness of training in improving pesticide knowledge in farmers [14,15].

This study has several strengths. First, the participants varied considerably in age, pesticide-related activities, experience with pesticide use, and type of farm. This diversity ensures that the safety climate estimates obtained are representative and generalizable. Second, a pre- and post-intervention design was used (D0 and D1 or D2), enabling us to discern training effects, as the individual risk factors remained constant. Third, despite the moderate sample sizes (*n* = 182 and *n* = 164), the major impact of the intervention studied (the *Certiphyto* training course) resulted in highly significant differences between the mean scores.

This study also had some limitations. First, our approach relied on participants to provide thoughtful and sincere responses, but we did not validate the responses recorded through field observations. We hoped that the voluntary nature of participation and anonymity would encourage honest and open feedback, thus ensuring the reliability of the survey results. Second, data regarding the fruit and vegetable crops cultivated by the course attendees were not collected during the survey, which prevented analyses by crop categories. Third, only a few factors that are potentially associated with safety climate change were considered. However, due to the significant effect size of the intervention, it is unlikely that residual confounding could account for the observed differences. Fourth, the training course was brief, lasting a maximum of two days, which means that only short-term effects could be demonstrated without making assumptions about eventual long-term effects.

We found evidence of increases in participants’ global safety climate scores and in each of the sub-scores after they completed the *Certiphyto* training course regardless of the cultivation type (tree fruit or fresh vegetable). It may appear improbable that a brief educational session could alter the work environment, which is mostly shaped by habits, procedures, and policies; however, our findings demonstrate that training can affect individual *perceptions* of the work environment by offering new knowledge and skills. The increases in scores (8.2% and 8.6% for global scores among fruit and vegetable producers, respectively), were unexpectedly substantial, leading to highly significant results. As anticipated, the dominant increases were observed in the dimensions of knowledge as well as communication and feedback, which was consistent with causal inference, as these topics represented the core of the training program. Conversely, farmers demonstrated some resistance to change in the rules and best practices dimension, which earned the smallest but still significant increase in scores (3.4% and 2.6% for the global score among fruit and vegetable producers, respectively). In this respect, introducing peer-to-peer learning could contribute to overcoming the slow adoption of sustainable cultural practices, as farmers could witness the benefits firsthand.

Increases in safety climate scores were generally unaffected by demographic or occupational characteristics, and the signs of the few significant covariate-by-time interactions moved in the expected direction. The most prevalent risk factor, already observed among cereal producers [18], was the type of certification. This factor was found to play a significant role in the global scores, and the dimensions of management commitment, communication and feedback, and knowledge. Score increases after the intervention were lower for farmers attending a certification refresher course. These attendees represented the most-trained farmers, who are known to display safe behavior in pesticide use [13]. The margin for improvement was therefore smaller for these individuals, as reflected by these negative covariate-by-time interactions. A negative interaction was also found for the participants’ position in farming activities. Farm workers experienced fewer benefits from the training course in three dimensions (communication and feedback, knowledge, and safety participation), as indicated by smaller increases in the corresponding sub-scores. This may reflect a lesser interest in these topics, which are meant to be dedicated to managers. One interaction specific to fresh vegetable cultivation is worth noting: individuals who worked on organic farms illustrated greater improvements in the dimensions of communication and feedback as well as knowledge. This could be a result of organic producers’ holistic approach, which integrates social, economic, and ecological factors and is enhanced by a network of cooperation with other farmers [31]. The few remaining covariate-by-time interactions seemed essentially anecdotal and could be attributed to chance findings.

All the studies considered in the two available reviews on the effectiveness of educational interventions were conducted outside of Europe, which prevents any comparison with the current study [14,15]. However, we have identified two European studies that have been released in the meantime. The first study is based on interviews with Greek farmers in areas where cotton is cultivated. However, the agricultural practices for cotton crops are very different from those observed in tree fruit or fresh vegetable cultivation [13]. The second study compares safety climate scales before and after the French *Certiphyto* training like the current study, but focuses on cereal farmers [18]. Overall, there were similar increases in mean scores, although a sex-by-time interaction was found in cereal production. Smaller improvements were observed in men compared to women for global score, communication and feedback, and knowledge scores. This consistency reinforces the importance of training and certification procedures for a more sustainable use of pesticides.

## Conclusion

Reducing pesticide exposure in agricultural activities remains a major challenge, particularly in productions that rely heavily on pesticides. This study demonstrates that the training provided to French fruit and vegetable farmers and farm workers enhances their perception of the safety climate. This confirms that the *Certiphyto* training is successful in ensuring that professionals have the necessary knowledge to implement sustainable agricultural practices.

However, as some sub-scores reveal only slight increases and the few significant determinants differ depending on the type of production, it may be relevant to upgrade the current training program. This would require involvement from stakeholders including farmers, farm workers, farm union representatives, ministry of agriculture officials, trainers, and professionals in occupational health and safety. The training program could be enhanced by adjusting both its content (e.g., tailoring it to different groups of producers) and structure (e.g., incorporating peer-to-peer training). Future research on this revised training initiative should document farmer’s behaviors towards pesticide use and include biological biomonitoring. The long-term effects of such pesticide safety training could then be evaluated in terms of safety climate, pesticide application practices, and pesticide exposure in the ever-evolving landscape of agriculture, with the ultimate goal of reducing risks to health and the environment.

## Supporting information

S1 Table French fruit producers dataset. (XLSX)

S2 Table French vegetable producers dataset. (XLSX)
